# Radiomics-based differentiation of lung disease models generated by polluted air based on X-ray computed tomography data

**DOI:** 10.1186/s12880-016-0118-z

**Published:** 2016-02-11

**Authors:** Krisztián Szigeti, Tibor Szabó, Csaba Korom, Ilona Czibak, Ildikó Horváth, Dániel S. Veres, Zoltán Gyöngyi, Kinga Karlinger, Ralf Bergmann, Márta Pócsik, Ferenc Budán, Domokos Máthé

**Affiliations:** Department of Biophysics and Radiation Biology, Semmelweis University, Tűzoltó utca 37-47, Budapest, H-1094 Hungary; CROmed Translational Research Centers Ltd., Baross utca 91-95, Budapest, H-1047 Hungary; Department of Radiology and Oncotherapy, Semmelweis University, Üllői út 78/A, Budapest, H-1082 Hungary; Department of Public Health Medicine, University of Pécs, Szigeti út 12, Pécs, H-7624 Hungary; Institute of Radiopharmaceutical Cancer Research, Helmholtz-Zentrum Dresden-Rossendorf, Dresden, D-01314 Germany; MedProDevelop Kft, Irgalmasok utcája 16, Pécs, H-7621 Hungary

**Keywords:** Fractal dimension, Radiomics, In vivo micro-CT, Air pollution, Lung disease

## Abstract

**Background:**

Lung diseases (resulting from air pollution) require a widely accessible method for risk estimation and early diagnosis to ensure proper and responsive treatment. Radiomics-based fractal dimension analysis of X-ray computed tomography attenuation patterns in chest voxels of mice exposed to different air polluting agents was performed to model early stages of disease and establish differential diagnosis.

**Methods:**

To model different types of air pollution, BALBc/ByJ mouse groups were exposed to cigarette smoke combined with ozone, sulphur dioxide gas and a control group was established. Two weeks after exposure, the frequency distributions of image voxel attenuation data were evaluated. Specific cut-off ranges were defined to group voxels by attenuation. Cut-off ranges were binarized and their spatial pattern was associated with calculated fractal dimension, then abstracted by the fractal dimension -- cut-off range mathematical function. Nonparametric Kruskal-Wallis (KW) and Mann–Whitney post hoc (MWph) tests were used.

**Results:**

Each cut-off range versus fractal dimension function plot was found to contain two distinctive Gaussian curves. The ratios of the Gaussian curve parameters are considerably significant and are statistically distinguishable within the three exposure groups.

**Conclusions:**

A new radiomics evaluation method was established based on analysis of the fractal dimension of chest X-ray computed tomography data segments. The specific attenuation patterns calculated utilizing our method may diagnose and monitor certain lung diseases, such as chronic obstructive pulmonary disease (COPD), asthma, tuberculosis or lung carcinomas.

**Electronic supplementary material:**

The online version of this article (doi:10.1186/s12880-016-0118-z) contains supplementary material, which is available to authorized users.

## Background

“Radiomics” is an approach currently recognized in biomedical image analysis as a tool to define a potentially diverse array of meta-data obtained from images using quantitative radiology image analytics. Some well-selected features of these meta-data can be informative of the health status of the imaged organ system and impact therapy decisions. Such therapy decisions are best taken early in the course of disease. Early therapy decisions have tremendous impact on quality of life in pulmonary diseases.

Fractals are often used to characterize non-Euclidean structures in biology [1]. Utilizing the scaling factor of statistically self-similar and non-overlapping subsets, fractal dimension can be computed [2] providing relevant information describing a structure’s complexity and homogeneity [3]. Fractal dimension represents, with certain limitations, the “less or more branching nature” of structures [4, 5] including the respiratory organ [6].

We sought to implement a fractal-based radiomics approach to X-ray computed tomography attenuation data without respiratory gating thereby averting the risk of losing relevant information. Analysis of fractal dimensions in specially binned non-gated X-ray computed tomography image patterns has been the method of our choice.

The currently accepted method of analysing pulmonary fractal dimensions of X-ray computed tomography attenuation data usually consists of segmenting parts of the lung such as the alveolar respiratory units, or pulmonary arteries and veins [7–10]. Al-Kadi and Watson [2] distinguished tumours and blood vessels based on their X-ray attenuation differences (using contrast material) to perform fractal dimension analysis on the image segments. The usually applied methods therefore provide the reader with a fractal dimension value for each of the tissue component segments of lung images. A usual outcome measure e.g. is the fractal dimension of lung arterial vasculature.

Our approach to radiomics has been greatly different from simply calculating fractal dimensions of segmented pulmonary tissue components (“dissected” vessels, bronchi etc.). The examination of fractal dimensions of ideally selected attenuation ranges in relative Hounsfield units (HU) may provide the foundation towards discovering additional hidden tissue features in integrative patterns of lung images instead. These fractal dimension calculations may perhaps detect small scale tissue alterations such as those caused by harmful environmental conditions. Our objective was to unveil possible correlations between air pollutant categories and specific features or patterns of damaged lungs. These features of small magnitude might not be evident in either custom visual X-ray computed tomography image analysis or in the calculation of segmented pulmonary tissue fractal dimensions.

We aimed at distinguishing between different air pollutant effects on the lungs via a radiomic approach with a clinically translatable mathematical algorithm. We preferred using non-gated X-ray computed tomography data. Non-gated data acquisition still contains effects of e.g. hindered chest or lung motion. In our analysis we intended to examine data features reflecting disease-related changes also in lung organ movements rather than anatomical relationships. Thus in our analysis method presented here simple non-gated X-ray computed tomography mouse chest scans have been acquired and evaluated by the calculation of fractal dimension of binary images. We binned voxel sets from each mouse chest X-ray computed tomography volume into numerous attenuation ranges in our study [1, 11], instead of pulmonary tissue-based image segmentation. Additionally we also averted the use of any contrast agent.

Generally speaking, (both in the “classic” and in our novel method), the result of fractal dimension calculation is a number corresponding to how often examined structures (dissected arteries and veins in “classical” methods and voxel 3D patterns with specific attenuation values in our approach) branch and/or fill the space within the chest. However, in our novel approach, the fractal dimension calculation method examines and depicts integrative binary images of lung voxels which are selected according to their attenuation values. We then aimed at the application of our algorithm to discriminate among groups of mice treated with different air pollutants in an early phase of their respective disease models.

## Methods

### Ethics Statement

The animal experiment was reviewed and approved by the local authorities (Committee on the Ethics of Animal Experiments of Semmelweis University, permit number: PMK ÉBÁI-XIV-I-001/29-7/2012) according to Hungarian animal protection laws in accordance with EU guidelines.

### Experimental animals

**Fig. 1:**
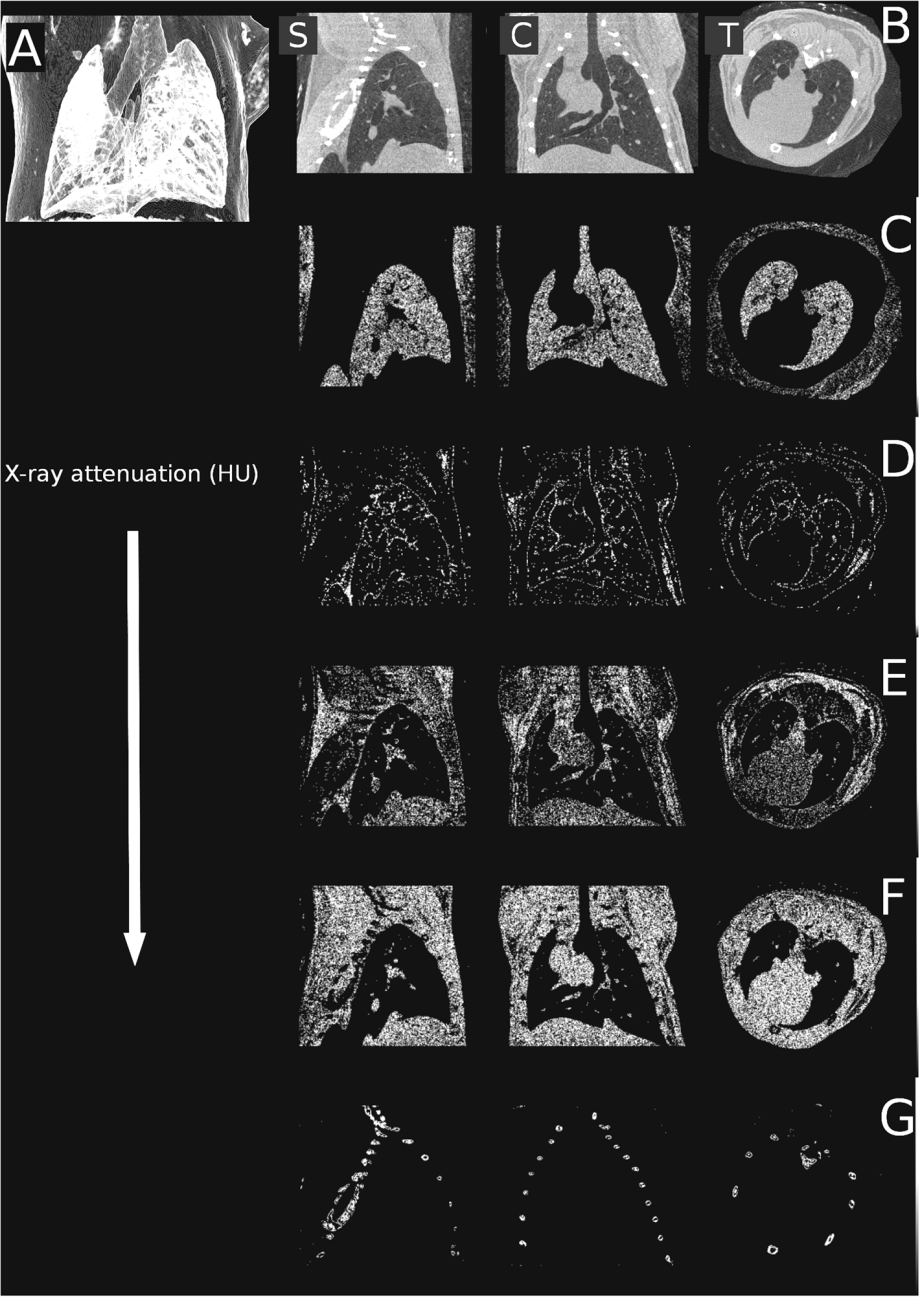
The reconstruction of the lungs of an untreated mouse shown with the sole intent of representing the attenuation profile of the chest. Sagittal (left **b**), coronal (center **b**), transaxial (right **b**) planes and minimum intensity projection (**a**). Certain attenuation ranges were abstracted from these slices in sagittal, coronal and transaxial planes. **c** −700 – -400 relative HU (lung parenchyma), (**d**): −100 – +200 relative HU (pleura, endothoracic fascia, epipleural fat and interlobar fissures), (**e**): +200 – +500 relative HU (respiratory- and heart muscles, diaphragm), (**f**): +500 – +800 relative HU (blood inside the vessels, aorta and heart, lymphatic fluid and interlobar fissures), and (**g**): +1400 – +3800 relative HU (bones)

Three groups of BALBc/ByJ female mice (6–8 weeks old, 18–22 g) were used in the context of our current research. Their exposure was performed in a plexiglass inhalator chamber (30 cm × 30 cm × 50 cm). One group (*n* = 5) was treated with inhalation of sulphur dioxide (SO_2_) gas 2 % v/v (SDO group). A second group (*n* = 5) was treated with air diluted, fresh mainstream cigarette smoke from ‘3R4F’ Reference Cigarettes (Kentucky Tobacco Research & Development Center, USA) mixed with ozone-air gas mixture (50 mg/h, 3.7 l/min dilution with air; SAO group). Cigarettes with a shortened filter only (approx. 2 mm) were smoked according to our protocol (1 puff/9 s of 3 s duration and 40 mL volume). The SAO group first received one 20 min long exposure, followed by two 20 min long exposures, and lastly, three 20 min long exposures on the additional remaining days. A control group (*n* = 6) was also used, treated with the inhalation of filtered and humidified (30–40 %), air under identical conditions (CON group). Treatment duration was 14 days, and then imaging was carried out within all groups. An untreated BALB/CyJ female mouse (Janvier, France) was imaged too, in serving the purpose of representation of the attenuation profile of the chest (Fig. 1).

### In vivo imaging

X-ray computed tomography information was collected using a NanoX-CT (Mediso Ltd, Hungary) cone-beam in vivo micro-CT imaging system (8 W power of X-ray source, 55 kV source voltage, 3.6x zoom) without using contrast material. It is important to emphasize the physics of cone-beam X-ray computed tomography acquisitions which represents specific technical considerations beyond the scope of our current paper and may distort the direct comparison of attenuation values of the same organs measured with other detection techniques, such as those applied in clinical slice-based X-ray computed tomography systems. Therefore, attenuation values are presented in relative Hounsfield units.

Reconstruction algorithm (Mediso Ltd.) utilizing the Feldkamp-filtered back projection was run on a 64 bit graphics processing unit (GPU). The reconstructed voxel sizes were 54 × 54 × 54 μm in one 370 × 370 × 370 voxel matrix, as region of interest (ROI) following our image acquisition. During data acquisition animals were constantly anaesthetized using a mixture of 2.5 % isoflurane and medical air, and their body temperature was maintained at 38 C. One image acquisition took 4.5 min. Non-respiration-gated data sets were acquired and further analysed.

Fan-beam technology, used earlier and extensively within clinical practice, has today largely been replaced by multislice detector technology. As a result, the difference between pre-clinical and clinical instruments is quickly disappearing and clinical systems are gradually becoming similar to cone-beam computer tomography instruments.

Sensitivity in small animal imaging may be worse when compared to the human counterpart. Voxel size is one tenth or twentieth of the human voxel size, and so the signal-to-noise ratio could be worse (as this ratio is inversely proportional to the third power of voxel volume), however, the measurement time is much longer. In summary, signal-to-noise ratio is better in human measurements, meaning our method will have an increased sensitivity when applied to human cases.

### Evaluation of X-ray attenuation histograms

**Fig. 2:**
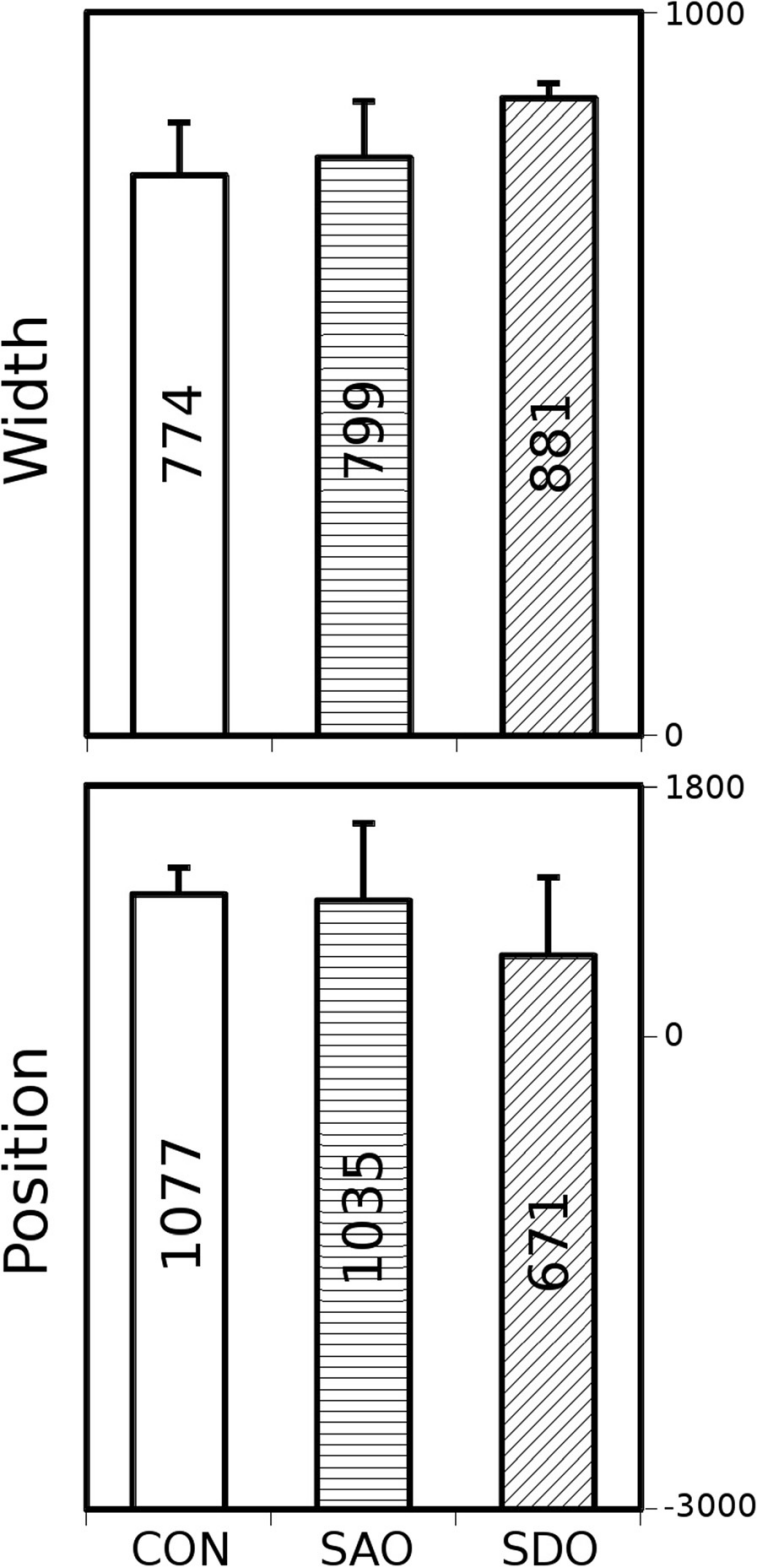
Width and position parameters (mean, SD) of attenuation histograms. CON, SAO, SDO groups

In the first step, our algorithm segmented the acquired 3D X-ray computed tomography volume reconstructions to contain only the whole lung volumes and to automatically distinguish between the lung tissue, chest bones and other tissues. The attenuation values of lung voxels were represented in a frequency distribution function, commonly referred to as a histogram [12] (data not shown). In the next step of evaluation, Gaussian curves were fitted by a minimum square algorithm (Gnuplot 4.4), featuring height, width and position. We calculated the means and standard deviations of positions and widths of the Gaussian curves of all examined groups (Fig. 2). The height is not presented on Fig. 2 as this parameter is dependent on width and area under histogram by definition.

### Characterization by fractal dimension analysis of voxels of various attenuation ranges

**Fig. 3:**
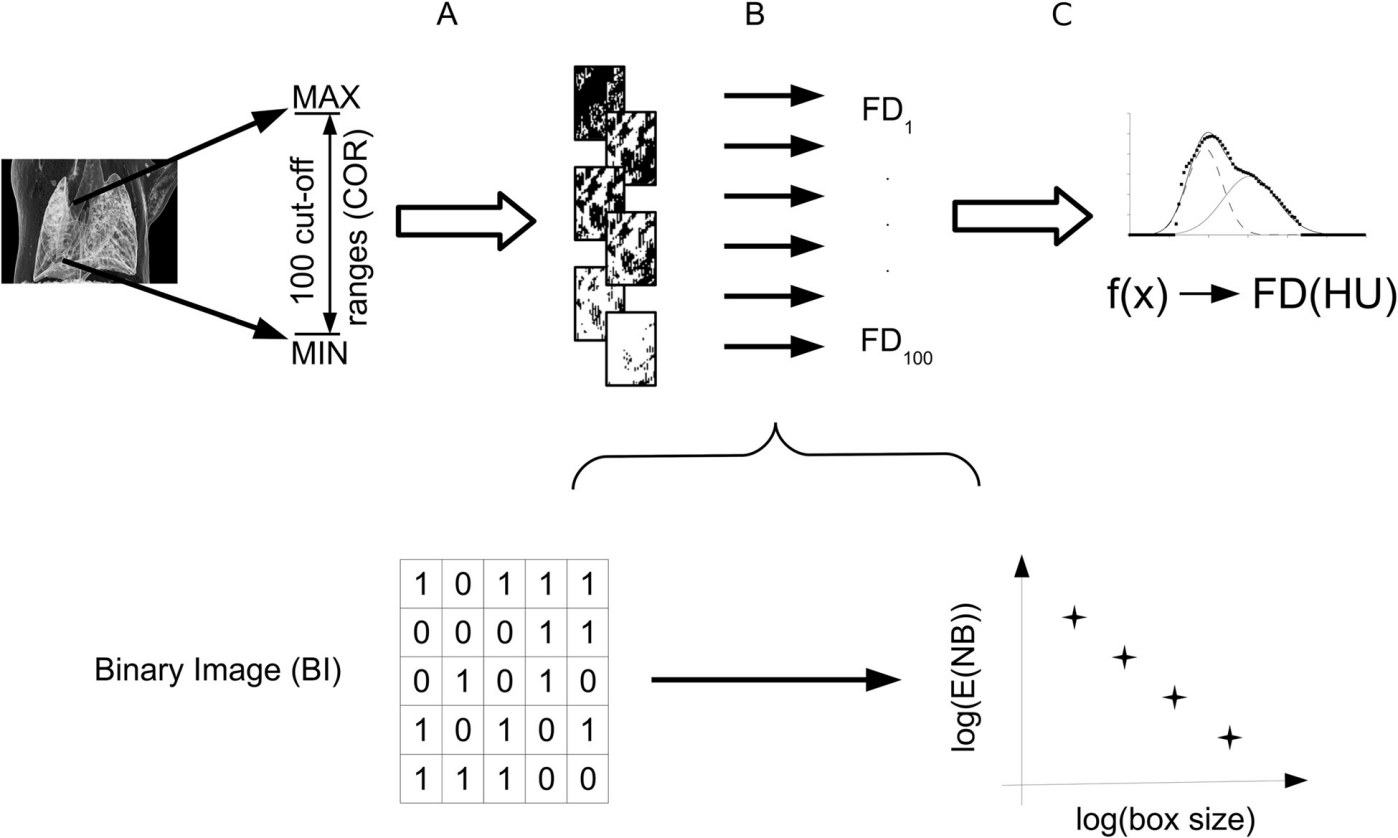
Representation of the five steps of data analysis. **a**: The entire attenuation range of the reconstructed chest area of animals was divided into 100 distinct cut-off ranges (Step 1). **b**: Binary images are generated (Step 2 and 3) and each such derived binary pattern was next associated with a calculated fractal dimension via box-counting algorithms (Step 4). **c**: The fractal dimension – cut-off range function plot (Step 5)

Final data analysis was performed in five steps (Fig. 3). The entire attenuation range of the reconstructed chest area of animals was divided into 100 distinct cut-off ranges. Once the attenuation value of a given voxel was within a certain cut-off range, the voxel was represented by “1”, or otherwise, “0”. This generated a binary image. Each such derived binary pattern was then associated with a calculated fractal dimension via box-counting algorithm [13]. The lung morphology was quantified by plotting the fractal dimension of all cut-off ranges in each given X-ray computed tomography 3D attenuation map. This plot is defined as the fractal dimension - cut-off range function.

Here we present the exact details of the mentioned five steps of data analysis with the purpose of demonstrating how the cut-off range associated fractal dimension data were achieved (Fig. 3).

Step 1. The whole attenuation range in the experiments was between −3,000 and +10,000 relative HU. The 100 distinct ranges of attenuation values were chosen by the Freedman-Diaconis rule [14]. A chosen range is defined mathematically as the standard cut-off range. Certain attenuation values ranging between the chosen higher and lower value were ranked into one cut-off range. The 100 cut-off ranges involved the whole scale of the voxel attenuations of all scans and one cut-off range contained 130 relative HU.
Step 2. The X-ray computed tomography images were partitioned into cubic voxels (size: 54 μm in a 370 × 370 × 370 voxel matrix). When the attenuation value of a given voxel was inside a certain cut-off range, then that voxel was associated with “1”, or otherwise with “0” in the representation of the cut-off range.
Step 3. This association step was repeated which resulted in a pattern of voxels with “1” and “0” for every cut-off range. These patterns as binary images were used in the next steps. The 100 binary images were derived from 100 previously defined cut-off ranges.
Step 4. The box counting algorithm [13] was used to calculate the fractal dimension number associated with each binary image. See additional details below in the sub-steps labelled a through d.
  - In this box-counting algorithm, the length of cubic boxes varied from 1 to 100 voxels. A given box was shifted from one position to another without overlapping in a certain binary image. The number of boxes containing at least one voxel with value “1” was summarized. Thereby, a number was calculated defined as the number of boxes of a given side length (NB).
  - The previous sub-step a) was repeated including the difference that the boxes are overlapped. Thus, NB results were produced derived from the shifted overlapped positions. A certain box size in a certain binary image produced two different NB results (from overlapping and non-overlapping boxes). From these two NBs, an average (E(NB)) was calculated and that value was used in further evaluation.
  - For each box size, both the a and b sub-steps were repeated. E(NB) was represented as the function of the box size in a certain binary image.
  - To determine fractal dimension, the function from sub-step c) was fitted by a power function. The exponent of the power law is the fractal dimension. To each binary image, a fractal dimension number was ordered and calculated utilizing this method.
Step 5. In this step, the cut-off ranges were associated with the calculated fractal dimension. At both the very high and very low cut-off range values, the fractal dimension number is zero, while near the middle values of cut-off ranges the associated fractal dimension number becomes nearly maximal.

**Fig. 4:**
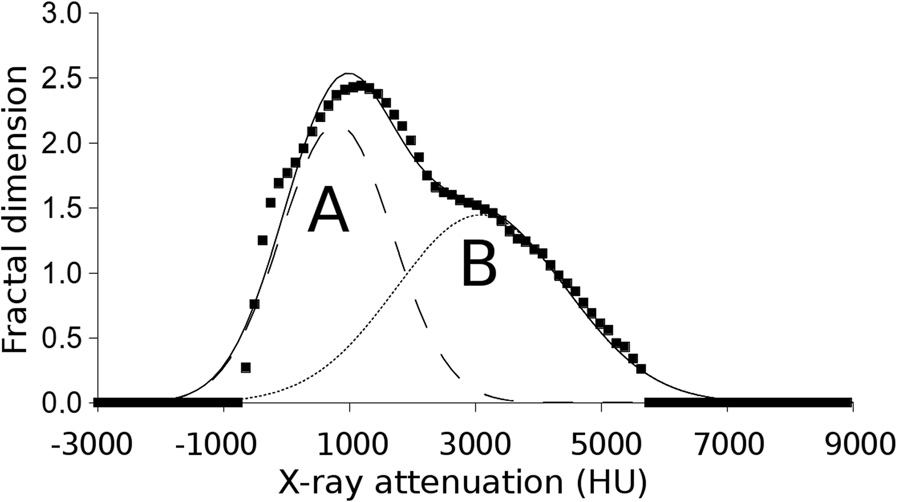
The fractal dimension – cut-off range function fitted by Gaussian curves “**a**” and “**b**”

The resulting fractal dimension - cut-off range function was fitted by two Gaussian curves (Gaussian curve “A” and “B”) using the so-called “least box square” algorithm. The outputs of our algorithm, the height, width and position parameters of these fitted “A” and “B” Gaussian curves, were calculated for all animals (Table 1). Lists these numerical features of the Gaussian curves for every group based on Additional file 1 (supporting data). The lungs of all three groups, SDO, SAO and CON, were characterized by these parameters (Fig. 4).

**Table 1:** Contains averages and standard deviations of all fitted parameters for all groups of animals

Average	SDO	SAO	CON
Height A	2,161	2,231	2,291
Height B	1,725	1,376	1,276
Position A	181	628	735
Position B	3951	3473	3776
Width A	1133	1036	1086
Width B	2496	1630	1562
STD			
Height A	0,049	0,104	0,042
Height B	0,080	0,063	0,090
Position A	386	540	217
Position B	110	647	277
Width A	61	165	169
Width B	161	169	135

**Fig. 5:**
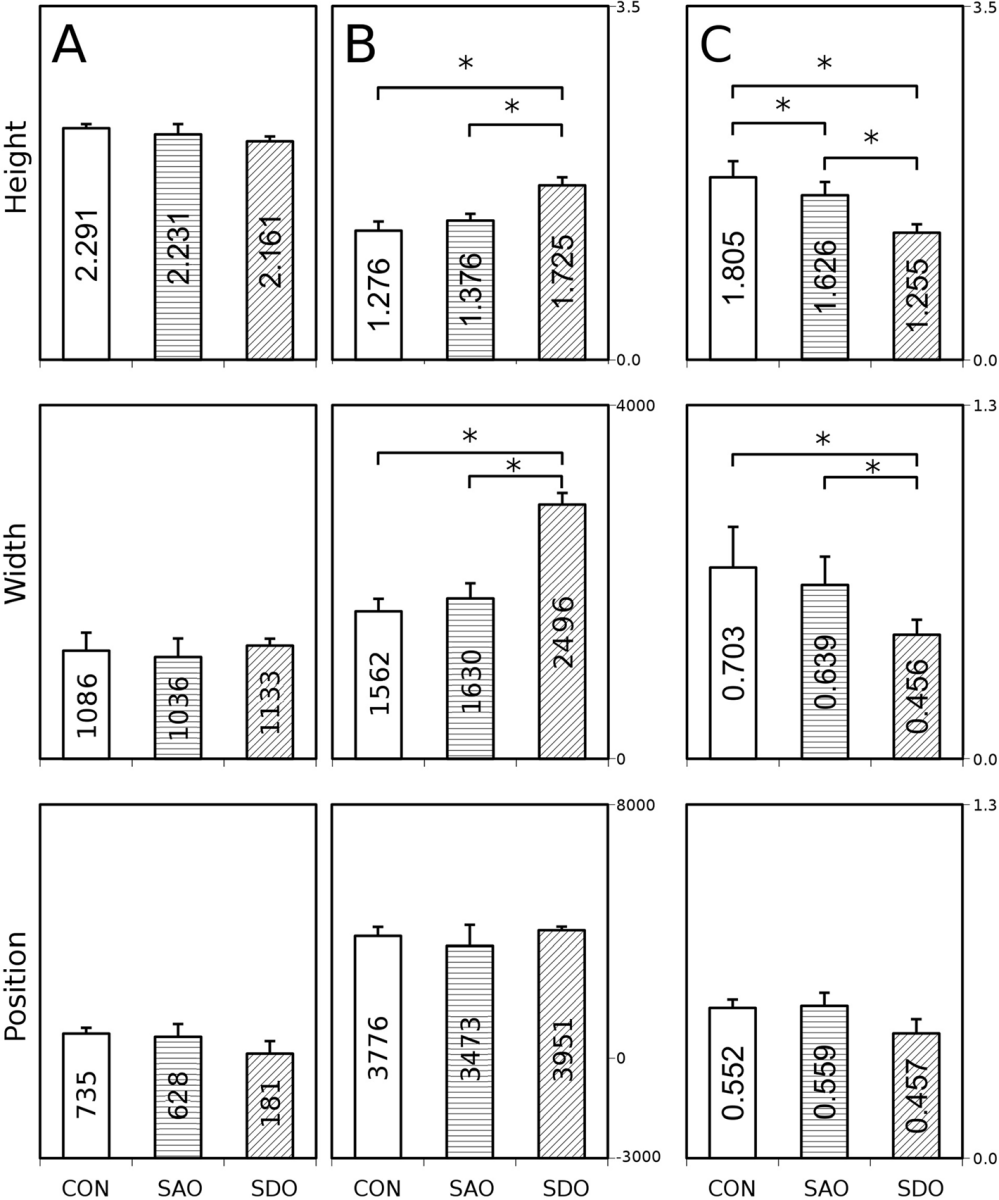
Calculated height, width and position parameters of the fractal dimension - cut-off range functions. **a**: Height, width and position parameters of Gaussian curve „A” (CON, SDO, SAO groups). **b**: Height, width and position parameters of Gaussian curve „B” (CON, SDO, SAO groups). **c**: The ratios of the relevant parameters of Gaussian curves „A” and „B” (CON, SDO, SAO groups). * *p* < 0.05, Kruskal-Wallis (KW) test with Mann–Whitney post hoc (MWph) test

Lastly, to further attempt significant discrimination of the groups, ratios of all three mean parameters derived from both “A” and “B” Gaussian curves were calculated. The C insets represent the ratios of “A” parameters divided by “B” parameters (where a certain “C” parameter is calculated by dividing the “A” parameter of a given group by the “B” parameter of the same group) (Fig. 5).

These steps were repeated to evaluate the images of each animal.

### Statistical analysis

Statistical analysis was performed using the nonparametric Kruskal-Wallis (KW) test for fitted parameters of groups (STATISTICA 7.0, Statsoft Inc., USA). Differences between all groups were evaluated by the Mann–Whitney post hoc (MWph) test (Figs. 2 and 5).

A chi-square test was used to test the reliability (*p* < 0.05) of fit of the histogram and the fractal dimension, or the cut-off range function (either fitted with one single or two independent Gaussian curves).

## Results

The mean values of width and position of the voxel density histograms demonstrate no significant differences between the three groups (Fig. 2).

The fractal dimension - cut-off range functions were evaluated by fitting them with Gaussian curves “A” and “B” (Fig. 4). These functions can be characterized by height, maximum position and width of the peak. The means of height, width and position of the “A” curve of the CON group do not differ significantly when compared to the SDO or the SAO group. The means of height, width and position are unchanged between the SDO and SAO groups, too (Fig. 5a).

The mean of height of “B” curve of the SDO group increased significantly when compared to the CON group (KW *p* = 0.002, MWph *p* = 0.036) and to the SAO group (KW *p* = 0.002, MWph *p* = 0.024), but not significantly when the CON group was compared to the SAO group (Fig. 5b top). The mean of widths of “B” curves of the SDO group increased significantly (KW *p* = 0.016, MWph *p* = 0.036) compared to the CON group, and also significantly when compared to the SAO group (KW *p* = 0.016, MWph *p* = 0.024), but not significantly when the CON group was compared to the SAO group (KW *p* = 0.016, MWph *p* = 0.429) (Fig. 5b middle). The means of maximum positions are not significantly altered between the SDO, SAO and CON groups (Fig. 5b bottom).

The difference between the ratios of height is significant if the SDO group is compared to the CON group (KW *p* = 0.005, MWph *p* = 0.0357), if the SDO group is compared to the SAO group (KW *p* = 0.005, MWph *p* = 0.024) and if the SAO group is compared to the CON group (KW *p* = 0.005, MWph *p* = 0.042) (Fig. 5c top).

The ratios of width of the SDO and CON groups (KW *p* = 0.021, MWph *p* = 0.036) and the SDO and SAO groups (KW *p* = 0.021, MWph *p* = 0.024) demonstrate a slight but significant difference (Fig. 5c middle), however, the difference between the SAO and CON groups is not significant.

## Discussion

Differences between parameters based on the relative HU--frequency histograms of the animals in the three groups, i.e. the differences between mean maximum positions and widths (Fig. 2) could be neither the basis of detection of an altered lung structure nor the categorization of air pollution exposure. Either the absence of gating or the reduced period of mouse model symptom production was likely the reason. Indeed, Sasaki et al. (2015) too could not distinguish the effect of cigarette smoke on lung tissue attenuation in comparison to the control using X-ray computed tomography with gating [15]. However, interestingly enough, a number of differences could be shown using our novel radiomics analysis method.

In our opinion, there are distinctly altered tissue features with respectively changed attenuation patterns in the different mouse models of air pollution related disease. However, the readout of these changes necessitates subtle differentiation and radiomics analysis methods in early phases of lung harm when symptoms are yet considerably subtle.

Severe lung diseases alter the inhalatory and exhalatory movements and these changes can be detected even in mild cases or after relatively short exposure to air pollutants (e.g., the slower exhalation in COPD is evident) [16, 17]. This may be paradoxically advantageous in our approach and likely will help in discerning exposure sources. The hindered motion in the smoke/air and sulphur dioxide groups presumably decreases or even negates spatial and temporal overlapping of different tissues in the same voxel caused by respiratory movement and finally contributes to the mentioned increase of width parameter in the exposed groups. This change in motion dynamics (probably due to inflammation, mucus build-up and entrapped air bubbles) may be an important part of the diagnosis and it is ignored when using respiratory gating. Notably, this finding suggests the fractal dimension- cut-off range function derived radiomic data might unveil some pathologic changes in lung diseases [3, 18]. Possibly the onset of disease as a result of exposure to air pollution could also be observed with our data analysis method.

The Gaussian curves of Fig. 5 display a different pattern of respiratory pulmonary motion in the exposed animals, possibly due to an increase in lung stiffness caused by pollutants.

The height of Gauss curve B is significantly increased in the SDO group compared to the other two groups (Fig. 5b top). We infer this change is attributable to the hindered motion of inflamed tissue.

Sulphurous gases are irritants and induce inflammation, bronchoconstriction and bronchitis resulting in an increase of mucus [3, 19]. Overproduction of mucus can form plugs which entrap air or temporally and partly obstruct the upper airways [20]. Airway clearance of mucus depends on the interactions between physical properties of the mucous gel, serous fluid content, and ciliary function, in addition to airflow [19]. Wagner et al. (2006) discovered in a Sprague–Dawley rat model that 80 ppm concentration of SO_2_ (besides overproduction of mucus) caused epithelial cells to lose their ciliae [21]. Nano-sized solid particles originating from fumes tend to accumulate in deeper airways and alveoli [22], as inflammatory agents increase water permeability and dilate cell volume thus thickening airway walls and resulting in the narrowing of the airways (in addition to minor mucus production which cannot be excluded). In our interpretation, this narrowing of the airways causes the different motion dynamics of this group.

The width parameter of the SDO group is significantly increased compared to the other two groups (Fig. 5b center). We believe this change is attributable to the hindered motion of inflamed tissue.

The number of voxels representing thickening airway walls is increased, caused by SO_2_ exposure often penetrating into deeper airways and inducing inflammation in the alveoli, leading to the appearance of fluid, derived from necrotic cells [19, 23]. Mucus plugs trap air inside the alveoli and lead to the formation of micro-sized bubbles [24, 25] inside the lung parenchyma. Indeed mucus production is an early response to increased amounts of air pollution [19]. In our interpretation, the increased number of voxels representing thickening airway walls causes the different shape of the fractal dimension - cut-off range function of this group.

Only a slight difference was observed between the means of height of Gaussian curve “B” of SAO and CON groups (Fig. 5b top). In addition to mean values of maximum positions (Fig. 5b bottom), the width parameter of the SAO group was compared to the control group (Fig. 5 middle), however it did not significantly change.

Theoretically speaking, emphysema-diseased areas within the lungs could occur caused by destroyed walls of airways and alveoli [11, 12, 18], however, it appears only in long term experiments [26].

In using the corresponding ratios of parameters of Gaussian curves “A” and “B” (Fig. 5c) and the heights and widths of “B” Gaussian curves (Fig. 5b), all three groups could be distinguished. We believe the difference between the ratios of parameters refers to the different proportion of various kinds of tissue damage caused by different air pollution agents.

The proportion of tissue alterations has a specific pattern in lung diseases of different origin. Altered tissue (e.g., increased mucus production in one model or increased presence of tissue microbubbles in another) was explored.

The mechanism of these changes affecting the respiratory movement remains unclear and warrants further research.

Data acquired in our study (Fig. 5b, c) proved a worthy basis for differentiating specific air pollution caused lung changes in the early stage by direct fractal dimension - cut-off range function pattern analysis. It could be hypothesized that molecular features and presence of mucus in smaller airways [19] and inflammation profile of lung tissues [27] contribute to our fractal dimension analysis-based results. In reference to published literature [19, 28], we postulate that the fractal dimension - cut-off range function calculated with our method may be used as an imaging biomarker. It could be effectively converted to both preclinical applications and clinical use in humans, ideally providing patients the benefit of early warning towards avoiding environmental risks. Additional benefits are expected in the proper treatment at the onset of symptoms of disease, and lastly, to prevent aggravation of disease and exacerbation of COPD and/or asthma bronchiale symptoms. Here, we highlight the importance of length of smoke exposure and genetic susceptibility [28] to emphysema [26], which may later develop and will be reflected in the reported imaging biomarker parameters [3, 18].

The radiomic analysis of the fractal dimension - cut-off range function may be useful in early diagnosis of both exposure to air pollution and lung diseases (such as COPD or asthma), containing information about both the molecular features and patterns of mucus in smaller airways [19] and the inflammation profiles of lung tissues [27]. We propose an increase in the number of structurally quantitative imaging biomarker research studies, towards early detection and follow-up of therapy of other pulmonary diseases, for example, cystic fibrosis, lung carcinomas [3] or tuberculosis [29].

In translational research, our most important goal is to develop methods in animal models which later may be used in clinical practice. In the case of our paper, the translatability of the method is dependent upon three aspects of it. The first is the usability of the algorithm in clinical practice, the second is the relation between anatomy sizes and reconstruction voxels, and the third is the applicability of the algorithm in clinical protocols.

The algorithm does not use specific data, as it only requires a 3D reconstruction, that is attenuation distribution. These data are also available in the clinical setting, so our algorithm proposed here can also be applied for clinical lung computed tomography volumes.

The human body is about 15–20 times longer than the body of the mouse and there is nearly the same difference between spatial resolution (voxel size in preclinical CT is 50 μm and in clinical CT is 500–750 μm). Generally speaking, anatomy size and voxel size change proportionally. Because of the partial-volume effect, though, true resolution does not reach voxel size calculated from reconstruction. It is important to note that the size of alveoli is invariable between species and is around 200 μm. Consequently, neither the pre-clinical nor the clinical instrument can visualize individual alveoli with suitable resolution, but in the case of the pre-clinical instrument, we see one alveolus in the adjacent voxels, whereas in case of the clinical instrument, we see more than one alveoli in one voxel.

Practically speaking, the proposed algorithm can be applied in the clinics and it is not necessary to change protocols, since the usual examinations and unchanged data acquisition chain should be followed by this new “off-line” analysis.

In summary, the use of the algorithm will be self-evident in clinical practice. Naturally, its diagnostic effectiveness needs to be assessed meticulously throughout different diseases.

## Conclusions

We discovered a novel diagnostic and disease characterization method providing results within a remarkably short disease model production time compared to the former 8–24 weeks needed to produce detectable lung tissue changes as described in published literature [30]. Additionally, as our method does not apply gating, it may contribute to the simplified and more cost-effective (through higher throughput) data analysis utilizing simple X-ray computed tomography scans in mouse experimental models. The implementation of our data analysis is straightforward and applicable in clinical image data sets and does not require additional hardware. As the early diagnostic potential of COPD-related lung and airway changes was shown here in data containing approximately clinical levels of noise, we remain convinced the translation and validation of our algorithm and data analysis in human clinical trials is warranted [3].

## Additional file

Additional file 1:
**Lists of the numerical features of the Gaussian curves for every mouse.** (DOC 36 kb)
