# Supplementary material for: Radiomics-based differentiation of lung disease models generated by polluted air based on X-ray computed tomography data
Source: BMC Med Imaging. 2016 Feb 11;16:14. doi: 10.1186/s12880-016-0118-z (PMC4750279; doi:10.1186/s12880-016-0118-z)
Supplement: Additional file 1: — Lists of the numerical features of the Gaussian curves for every mouse. (DOC 36 kb) [file 12880_2016_118_MOESM1_ESM.doc]

**Supporting information**

Table 2. Table contains the individual values of all fitted parameters for all animals.

|  | **SDO** | **SAO** | **CON** |
| --- | --- | --- | --- |
|  | **(n=5)** | **(n=5)** | **(n=6)** |
| **Height A** | **2,186** | **2,196** | **2,261** |
| **Height B** | **1,706** | **1,341** | **1,278** |
| **Position A** | **55** | **256** | **434** |
| **Position B** | **4045** | **3068** | **3784** |
| **Width A** | **1103** | **939** | **1228** |
| **Width B** | **2556** | **1708** | **1678** |
|  |  |  |  |
| **Height A** | **2,191** | **2,374** | **2,235** |
| **Height B** | **1,656** | **1,378** | **1,296** |
| **Position A** | **-127** | **1346** | **707** |
| **Position B** | **3830** | **4344** | **3543** |
| **Width A** | **1092** | **983** | **960** |
| **Width B** | **2619** | **1666** | **1686** |
|  |  |  |  |
| **Height A** | **2,105** | **2,148** | **2,340** |
| **Height B** | **1,812** | **1,447** | **1,114** |
| **Position A** | **613** | **796** | **908** |
| **Position B** | **3978** | **3088** | **4093** |
| **Width A** | **1203** | **883** | **1290** |
| **Width B** | **2314** | **1359** | **1348** |
|  |  |  |  |
| **Height A** | **2,111** | **2,302** | **2,290** |
| **Height B** | **1,804** | **1,290** | **1,374** |
| **Position A** | **566** | **791** | **675** |
| **Position B** | **4061** | **3978** | **3358** |
| **Width A** | **1193** | **1306** | **930** |
| **Width B** | **2335** | **1605** | **1455** |
|  |  |  |  |
| **Height A** | **2,210** | **2,134** | **2,282** |
| **Height B** | **1,645** | **1,422** | **1,338** |
| **Position A** | **-205** | **-47** | **1051** |
| **Position B** | **3841** | **2884** | **3943** |
| **Width A** | **1072** | **1070** | **914** |
| **Width B** | **2658** | **1811** | **1633** |
|  |  |  |  |
|  |  |  |  |
| **Height A** |  |  | **2,3392** |
| **Height B** |  |  | **1,2565** |
| **Position A** |  |  | **635** |
| **Position B** |  |  | **3934** |
| **Width A** |  |  | **1194** |
| **Width B** |  |  | **1570** |
